# Jung’s “Psychology with the Psyche” and the Behavioral Sciences

**DOI:** 10.3390/bs3030408

**Published:** 2013-07-18

**Authors:** Raya A. Jones

**Affiliations:** School of Social Sciences, Cardiff University, Glamorgan Building, King Edward VII Avenue, Cardiff CF10 3WT, UK; E-Mail: JonesRA9@cardiff.ac.uk; Tel.: +44-29-2087-5350; Fax: +44-29-2087-4175

**Keywords:** Carl Gustav Jung, science* versus* art debate, history of psychology

## Abstract

The behavioral sciences and Jung’s analytical psychology are set apart by virtue of their respective histories, epistemologies, and definitions of subject matter. This brief paper identifies Jung’s scientific stance, notes perceptions of Jung and obstacles for bringing his system of thought into the fold of the behavioral sciences. The impact of the “science* versus* art” debate on Jung’s stance is considered with attention to its unfolding in the fin de siècle era.

## 1. Introduction

To say that there is no place for analytical psychology in the behavioral sciences, does not mean that Jung’s work has no intrinsic value or relevance elsewhere. Insofar as “behavioral sciences” denotes traditional modern psychology, analytical psychology may provide at best an Archimedean vantage point from which to critique it. Jung took that attitude. The “modern belief in the primacy of physical explanations has led … to a ‘psychology without the psyche,’ that is, to the view that the psyche is nothing but a product of biochemical processes,” he contended, and called for summoning up “courage to consider the possibility of a ‘psychology *with* the psyche,’ that is, a theory of the psyche ultimately based on the postulate of an autonomous, spiritual principle” ([1], par. 660–661). Conversely, analytical psychology could be critiqued from the standpoint of the behavioral sciences, especially in terms of its methodology. 

Jung was making his point in 1931. Twenty-first century behavioral sciences have moved on from the psychologies he was criticizing. Yet, there remains the disparity he noted. On the one hand, sophisticated mathematical models applying dynamical systems theory, along with insights from brain imaging studies, have revitalized the interrelated notions of complexity and emergence. On the other, the trend has not resulted in a turn to holistic epistemology (on the contrary, much of it reinforces reductionism). While some contemporary Jungian analysts are attuned to conceptual trends in science (e.g., [2]), science is not attuned to the concerns of analytical psychology. The excitement about “embodied embedded cognition” is not without controversy in contemporary cognitive neuroscience (see, e.g., [3]); but those debates invariably concern the objective living body, not the subjectivity of the lived-in body. 

Pursuits of knowledge in analytical psychology and in the behavioral sciences are set apart by virtue of their respective histories, epistemologies, and definitions of subject matter [4,5,6], as summarized in this communication.

## 2. How did Modern Psychology Lose the Psyche

Jung begins his essay “On the Nature of the Psyche” with a historical review [7]. Up to the seventeenth century, psychology consisted of numerous doctrines concerning the soul, but thinkers spoke from their subjective viewpoint, an attitude that is “totally alien” to the standpoint of modern science ([7], par. 343). Incidentally, the German word *Seele* (soul) is usually translated into English as “psyche” when Jung writes about his own theory, perhaps because to Anglophone ears of the mid-twentieth century “psyche” sounded more scientific than “soul”. Jung’s point was that in the past philosophers’ theorizing was based on a naïve belief in the universal validity of their subjective opinions. Having reviewed the objectivity of modern science as an improvement upon pre-Enlightenment thinking, he comments that we can never remove ourselves from the subjective situation: “every science is a function of the psyche, and all knowledge is rooted in it” ([7], par. 357). Psychology as a science thus finds itself in an acute paradox, for “only the psyche can observe the psyche” ([8], par. 384). 

Spelling out the absurdity of the mind trying to observe itself, Comte had relegated psychology to a prescientific stage, and contended that psychologists mistook their own fantasies for science [9]. In Comte’s “metaphysical” stage, the supernatural beings of the primitive stage are replaced with “abstract forces, veritable entities (that is, personified abstractions) inherent in all beings, and capable of producing all phenomena” ([9], p. 26). This characterization readily applies to notions of libidinal forces and to innate archetypes. In the “scientific” stage, according to Comte, the mind applies itself to the study of the laws of phenomena, describing their invariable relations of succession and resemblances. The behavioral sciences have aspired towards the positivist ideal. The discipline’s historiography dissociates psychology not only from philosophy but also from “metaphysical” depth psychology. 

Comte was referring to the long history of psychology as a natural science. Philosophers following the Aristotelian tradition regarded the science of the mind as belonging to physics (*i.e.*, the science of nature). However, in the twentieth century, psychology became equated with quantitative experimental methodology, and this “scientific” character was contrasted with the “metaphysical” character of its earlier namesake [10]. Textbooks written by psychologists typically describe psychology as coming into being by virtue of its split from philosophy when Wundt opened the first laboratory in Leipzig in 1879. Between 1880 and 1920, American psychologists waged a battle against spiritualism and psychic research in their attempt to define boundaries for their new discipline [11]. William James started his essay “A Plea for Psychology as a ‘Natural Science”’ with the contention that although psychology was “hardly more than what physics was before Galileo … a mass of phenomenal description, gossip, and myth,” it nonetheless included enough “real material” to justify optimism about becoming “worthy of the name of natural science at no very distant day” ([12], p. 146). Four decades later, Lewin admitted that the battle “is not by any means complete,” but optimistically opined that the “most important general circumstances which paved the way for Galilean concepts in physics are clearly and distinctly to be seen in present-day psychology” ([13], p. 22). To date, a Galilean revolution has not happened. Yet, as Coon put it, psychology “has never recovered from its adolescent physics envy” ([11], p. 143). Although psychologists today seldom compare their science to physics, they tend to locate it within the natural sciences. For instance, Fuchs and Milar trace the origins of psychology to physiology (not philosophy) and its branching into psychophysics, and then through behaviorism to cognitive psychology [14]. 

Any telling of history is selective, biased in some way; and the bias serves an agenda. Costall exposes “a comprehensive and highly persuasive myth” about the origins of scientific psychology ([15], p. 635). He notes that according to most textbooks, psychology began as the study of mind based on the introspective method (associated with Wundt’s laboratory). In reaction to the unreliability of that method, behaviorism redefined psychology as the study of behavior, based on experimentation. In reaction to the bankruptcy of behaviorism, the cognitive revolution restored the mind as the proper subject of psychology, but now with the benefit of the rigorous experimental and statistical methods developed by the behaviorists—a storyline that has the structure of Hegelian thesis-antithesis-synthesis. Revisiting the early literature, Costall demonstrates that all three stages of this history are largely fictional. Moreover, “the inaccuracies and outright inventions of ‘textbook histories’ are not just a question of carelessness. These fictional histories help convey the values of the discipline, and a sense of destiny” ([15], p. 635). 

The psychoanalytic movement has been written out of that history and destiny (Costall does not mention it). However, in Jung’s time and place, “medical psychology” was more separate from the then-new science of psychology than present-day clinical psychology is from the behavioral sciences. In late-nineteenth century German universities, vested interests of influential professors played a key role in the designation of experimental psychology to the natural sciences [16]. Dilthey regarded psychology as belonging in the humanities on grounds that it concerns inner experience [17]. Drawing a contrast between the outer experience of nature (which is presented as phenomenal and in isolated data) and the inner experience of psychic life, which is holistically presented as a living active reality, Dilthey argued that for psychology to imitate a method that was successful in the natural sciences would involve treating an interconnected whole as if it were merely an assemblage of discrete entities. It would mean overriding descriptions of the subjectively lived experience in favor of the hypothetico-deductive method [18]. This argument has lost out in university departments; but it is implicitly sustained by analytical psychology to date.

## 3. Jung’s Scientific Stance

Foucault attributed the creation of the modern “soul” to historical conditions set in motion in the eighteenth century. Concepts such as psyche, subjectivity, personality, consciousness,* etc.*, were created so as to carve out domains of analyzing the post-Enlightenment soul, building upon it “scientific techniques and discourses, and the moral claims of humanism” ([19], p. 30). The moral claim is implicit in Jung’s statement, “We doctors are forced, for the sake of our patients, … to tackle the darkest and most desperate problems of the soul, conscious all the time of the possible consequences of a false step” ([20], par. 170). Yet, it is a paradox of modernity that when we seek to apply scientific techniques and discourses, the soul—the seat of subjectivity—vanishes.

Jung was a man of science by virtue of being a medical doctor, but he was not a scientist. He averred that unlike experimental psychology, analytical psychology does not isolate functions and then subject them to experimental conditions, but is “far more concerned with the total manifestation of the psyche as a natural phenomenon” ([20], par. 170). To him, the totality includes the unconscious as well as conscious mind. Being centered on the unconscious characterizes analytical psychology as a psychology with the psyche; and this characterization means that it would “certainly not be a modern psychology,” since “all modern ‘psychologies without the psyche’ are psychologies of consciousness, for which an unconscious psychic life simply does not exist” ([1], par. 658). 

However, his psychology does not merely state that an unconscious exists. It is premised on the notion that its existence can be demonstrated through observations of its effects. In this regard, his psychology is modern. It subscribes to the worldview—not the method—of modern science. As Weber put it in 1918, “The fate of our times is characterized by rationalization and intellectualization and, above all, by the ‘disenchantment of the world”’ ([21], p. 155); (see [22] for a historian’s account of this worldview). The model of the psyche that Jung was formulating in the same era could be viewed as an attempt to rationalize and intellectualize the enchantment of the world in myths, beliefs in the supernatural, and so forth. 

Jung unwaveringly professed a scientific stance, as did Freud. Making a case for psychoanalysis as a science, Freud defined *Weltanschauung* (worldview) as “an intellectual construction which solves all the problems of our existence uniformly on the basis of one overriding hypothesis” ([23], p. 195). Unlike religion, the *Weltanschauung *of science does not provide final answers. It “too assumes the *uniformity* of the explanation of the universe; but it does so only as a program, the fulfillment of which is relegated to the future” ([23], p. 196). Jung took a more categorical view: “A science can never be a *Weltanschauung *but merely a tool with which to make one” ([8], par. 731). Therefore “Analytical psychology is not a *Weltanschauung *but a science, and as such it provides the building-material … with which a *Weltanschauung *can be built up” ([8], par. 730). 

From the standpoint of behavioral sciences, depth psychology is a *Weltanschauung *that purports to solve all the mysteries of mind and behavior on the basis of one overriding (and irrefutable) hypothesis; namely, there is an unconscious mind. Could the unconscious be an object for scientific study? Such an object must exist independently of any description or interpretation of it and potentially be knowable in its entirety. Jung recognized the problems inherent in applying those criteria to the study of the psyche. Modern psychology “does not exclude the existence of faith, conviction, and experienced certainties of whatever description, nor does it contest their possible validity,” he pointed out, but “completely lacks the means to prove their validity in the scientific sense” ([24], par. 384). The dilemma stems from a mismatch between what we may want psychology to do for us (explain matters of faith,* etc.*) and what the scientific method permits us to do. 

The history of psychology in general could be viewed as an ongoing struggle with that dilemma. Often the “solution” has been to construe what Jung regarded as expressions of the psyche as being epiphenomena of either brain processes or language. As William James vividly put it, scientific thinking regards our private selves like “bubbles on the foam which coats a stormy sea … their destinies weigh nothing and determine nothing” ([25], p. 495). Yet nevertheless there is the reality of an “unshareable feeling which each one of us has of the pinch of his individual destiny,” a feeling that “may be sneered as unscientific, but it is the one thing that fills up the measure of our concrete actuality” ([25], p. 499). Jung could be viewed as endeavoring to formulate a system of concepts towards the systematic description of how that unshareable feeling becomes shareable—not only with other people, but first and foremost with one’s conscious self. It becomes accessible to conscious reflection through spontaneous symbolic representations of subjective states, Jung tells us throughout his works. 

## 4. Perceptions of Jung from the Standpoint of Scientific Psychology

Jung engaged with matters that were central to the formation of psychology as a modern science in the early twentieth century [26]. His early theory of the complexes, supported by the word association tests [27], accorded well with the experimental psychology of the day. Piaget still engaged with Jung’s theory in 1946 [28]; but by then Jung had fallen from grace in his home discipline, psychiatry. 

A browse through archives of *Nature *is illuminating. In a 1942 book review, the reviewer derogatorily labeled the Jungian approach a mystical psychology [29]. While applauding Jung’s early theory of the complexes as “scientific as any made before or since” in psychiatry, he reflected that Jung subsequently “abandoned his clinical work and most unfortunately started upon the study of religions and myths,” having “forsaken science for religion” ([29], p. 622). The critic misconstrued what Jung was doing. Jung was trying to explain religion scientifically. Nevertheless, after the word association experiments, the way Jung develops his ideas is not recognizably *science* as scientists know it. Consequently, even sympathetic critics were ambivalent about how to assess Jung’s contribution to science. In a 1954 review for *Nature*, Westmann commented that the book in focus (a collection of Jung’s writings) “shows the fundamental weakness of Jung’s psychology, which by having no fixed scheme appears to be full of contradictions and paradoxes; but this weakness is at the same time a sign of his greatness” ([30], p. 842). He elucidates by citing Heraclitus’s adage that you cannot step twice into the same river, and averring that “the life in the psyche manifests itself thus” ([30], p. 842). Talking of “life in the psyche” as taken-for-granted locates the speaker in the historical moment when the peculiarly modern Western conception of the self as an atomic unit was at its zenith. That conception has led to postulations of a universal mental structure as a necessity of nature. Jung reasoned, “Just as the human body represents a whole museum of organs, with a long evolutionary history behind them, so we should expect the mind to be organized in a similar way” ([31], par. 522). And yet, this inner structure is in constant flux like the proverbial river. 

Despite the proliferation of Jungian books in the second half of the twentieth century, there are no more reviews of such books in *Nature* after 1961 [32]. Readers of *Nature* are no longer expected to be interested in a mystical psychology. Contemporary scholars who study Jung are far more likely to be based in the humanities than in the behavioral or social sciences. 

Analytical psychology has been thoroughly removed from the scientific gaze. While there are sound reasons for dismissing claims that analytical psychology is scientific [5,6], there are not-so-good reasons, based in ignorance and misconceptions, for dismissing Jung. “We American psychologists are brought up to think of Jung as a mystic” ([33], p. 34). This applies also to British psychologists; or, rather, we have been brought up to think of Jung as a non-entity. In a typical syllabus, Jung features as a historical footnote to Freud. The Freudian story, which depicts Jung as a dissenting disciple, persisted after the behaviorists had debunked Freudianism. It was retained after behaviorism had given way to cognitivism. By the time that social constructionist critics of cognitivism appeared on the scene, Jungianism was too remote even to criticize. Meanwhile Freud was rediscovered, partially reinvented, by luminaries of postmodernism, and consequently arrived also in some variants of postmodern psychology. Jung remains excluded. Psychologists’ heightened moral and political sensitivities coincided with highly publicized allegations of Jung’s Nazi sympathies and anti-Semitism. The allegations are mostly unfounded [34,35], but the scandal has placed Jung off-bounds: “For political reasons I cannot allow myself to read Jung with pleasure,” stated Billig ([36], p. 6). 

Reading Jung is difficult with the best of will. The vast sweep of his eclectic knowledge results in verbose density and opacity. Navigating his voluminous writings inevitably means selecting threads of personal interest. Hence, Jung speaks differently to different readers. While there are books that reliably disseminate Jungian theory at a basic level, any simplification forfeits what historian Pietikäinen has aptly described as the kaleidoscopic nature of Jung’s psychology [37]. Many Jung-oriented publications have little in common with each other, and some have a dubious relation to Jung’s own work. There is “a profusion of ‘book-length commercials of Jungian therapy” and “pseudo-religious apologetics” ([37], p. 27). There are also works of academic excellence in analytical psychology; but their content tends to be too esoteric for the uninitiated. All that does not help to make Jung’s work a respectable pursuit for a behavioral scientist. 

## 5. Obstacles to Bringing Jung into the Fold

In and out of academia, “Jung” has become a kind of brand name that can be stamped on a variety of products. Since Jung regarded himself as first and foremost a psychologist, it is ironic that his work is appreciated by psychologists least of all. For the “typical” psychologist, the above barriers to engaging with Jung’s work are compounded by bafflement about what he was doing exactly. Readers of Jung schooled in the humanities may recognize a hermeneutic approach in his interpretation of myths, ancient scripts, and patients’ fantasies and dreams. Traditionally trained academic psychologists are not attuned to such methods. It is not clear how Jung gets from observation to theory. His transition from observing recurrent motifs in clinical and mythological material to a full-blown theory of archetypes is too rapid. He seems to be reading into the material his own expectations about the structure and dynamics of the psyche. Jung’s hypotheses must be taken on faith. Believers see the evidence everywhere, and seem to understand the task of empirical research as a matter of compiling catalogues of instances. It is not the logic of scientific discovery (cf. [38]; see [4,5,6] for an expanded discussion).

Jung talked the talk but didn’t do the walk. For most psychologists, it is primarily the praxis of psychological inquiry that differentiates it from other disciplines that also investigate mind and behavior. To some psychologists, it is not just any methodology but specifically the hypothetico-deductive method that makes it a *science*. Not all psychologists adhere to it in practice; but historically that classic ideal has dominated the behavioral sciences. The hypothetico-deductive method had been proposed by William Whewell in the nineteenth century, though it was Popper who has given it its best-known articulation [38]. In the 1930s, Popper contested the then-prevalent viewpoint associated with logical positivism, which regarded inductive reasoning as the basis for scientific inquiries. Induction proceeds from an initial explanation of some observations to its confirmation by collecting further empirical examples. This epistemological sin can be found in Jung’s progression from (a) observing recurrent motifs in dreams, visions, myths,* etc.*, through (b) theorizing those as archetypal manifestations, to (c) seeking to conform the existence of archetypes by observing more instances of the same. 

Despite Jung’s scientific stance, it is difficult to assimilate his ideas into the behavioral sciences not only due to how he went about validating them but also due to a lack of obvious connections with the ongoing preoccupations of the behavioral sciences. Even within Jungian circles, it is far from clear what “archetype” really means—lively debates continue to present day—partly because Jung’s own ideas changed over time [39]. Brooke attributed the difficulties that “psychologists of other persuasions” have with the concept to the fact that “archetypes seem mysterious, deep, remote, frightening, and enchanting, and thinking about them remains equally murky and ambivalent” ([40], p. 157). From my position as a non-Jungian psychologist, the problem is not necessarily the murkiness of the concept. There is little certainty at the cutting edge of science. If the concept were to excite scientists, its ramifications would have been explored. Rather, it is the *point* of postulating archetypes in the first place which eludes us “psychologists of other persuasions”. The very postulation seems redundant, a solution to a non-existing problem, an answer to a question that nobody else is asking. 

## 6. Science *versus* Art

The concept of archetypes failed to interest behavioral scientists, but has long fired the imagination of artists and literary writers. Jung’s theory is a powerful narrative. It might be correct in the way that a poem or a literary novel is correct; that is, as a whole coherent unto itself, all its elements in perfect relation to each other. A poetic gestalt-image impacts upon us aesthetically and emotionally irrespective of the factual veracity of its content. Whereas science seeks to establish objective truths about the world (and human nature) by narrowing down rival interpretations, the poetic process creates subjective truths through the multiplicity of overlain images and subjective connotations. 

Jung uses a similar strategy (cf. [41]). His hypotheses are speculative explanations—not testable predictions à la Popper—and he builds them by piling examples upon examples. Making a similar point, Hillman commented that Jung uses the word “empirical” to refer to a subjective process within him: “The empirical event—the solar-phallus image in a patient—releases a movement in the mind setting off a hypothesis … as a poem may start in a concrete perception”; and like a poet, “Jung returns ever and again to the concrete world of perceptions (cases, dreams, religious fantasies, ancient texts)” ([42], p. 32–33). 

Jung struggled with the incommensurability of science and art. In a talk on poetry, he asserted his standpoint as a scientist by endorsing the view of the two as mutually exclusive: “Art is by its very nature not science, and science by its very nature is not art” ([43], par. 99). The conflict came to his awareness in a typically Jungian manner, through a fantasy generated by his unconscious [44]. In 1913, whilst writing down disturbing fantasies he was having, he wondered, ‘“What is this I am doing, it certainly is not science, what is it?”—and a voice from nowhere told him it was art, a suggestion he strongly resisted though conceding that “obviously it wasn’t science” ([44], p. 42). The ambivalence carries across to his formal exposition of his theory. Analytical psychology is premised on the hypothesis that the psyche is an autonomous reality commanding specific energy. Yet such hypothesis “has its disadvantages for the scientific mind,” Jung comments; and continues, “In accordance with my empirical attitude I … prefer to describe and explain symbol-formation as a natural process” ([45], par. 338). His preference discloses a *language game *in Wittgenstein’s sense (cf. [46]). Language games are not “games” but profoundly shape attitudes and perceptions. In Jung’s milieu, the language game of science empowered those who came up with theories using words such as instincts, evolution, and energy; and eschewed words such as spirit. Jung labored to disengage his theorizing from religious mystification, seeking instead to explain all psychological phenomena as based in natural processes. 

## 7. Conclusions

Jung’s theory feels as true to some because it sounds scientific; to others it feels as false because it only *sounds* like that [5]. This seems like a deadlock of opinions. Instead of pinning the merit of Jung’s legacy on a categorical judgment of scientific/non-scientific status, it may be best to evaluate it in terms of applicability. We should ask, for whom and in what context does it serve particular purposes, and whether those would be served by the scientific method. 

The appeal of the Jungian approach in psychotherapy is evident in the worldwide success of the movement, but the clinical utility of particular concepts or techniques is not the same as their potential for generating hypotheses that scientists may explore in pure basic research. As seen, Jung himself made a categorical distinction between analytical psychology and experimental psychology [20]. Elsewhere, I revisit the implications of the differences between the practitioner’s ethic and the scientist’s ethic for analytical psychology and other contemporary approaches to the self [47]. 

Analytical psychology is not monolithic. It has its factions, and those too continuously evolve. Nevertheless, in all its versions, it concerns the holistic inner experience. It provides a way of thinking about and working with inner experiences. Hence, to echo Dilthey, it cannot be adequately served by methods of the natural sciences. Conversely, analytical psychology cannot readily serve a purpose in the behavioral sciences (in my view). It is clearly not a science of behavior in the way that the behaviorists have envisaged it. It is not a science of the mind in the way that cognitive science has been. By labeling it a psychology *with *the psyche, Jung implicitly positions its practitioner—not as someone who detachedly studies something called a psyche—but as someone trained to apply his or her own psyche as a tool towards trying to fathom how human beings attune themselves to own existence. 

## References

[1] Jung C.G. (1931). Basic Postulates of Analytical Psychology. The Collected Works of C. G. Jung.

[2] Cambray J. (2006). Towards the feeling of emergence. J. Anal. Psychol..

[3] Van Dijk J., Kerkhofs R., van Rooij I., Haselager P. (2008). Can there be such a thing as embodied embedded cognitive neuroscience?. Theory Psychol..

[4] Jones R.A. (2007). Jung, Psychology, Postmodernity.

[5] Jones R.A. (2014). Vicissitudes of a Science-Complex. Jung and the Question of Science.

[6] Jones R.A. (2003). On innatism: A response to Hogenson. J. Anal. Psychol..

[7] Jung C.G. (1954). On the nature of the psyche. The Collected Works of C. G. Jung.

[8] Jung C.G. (1927). Analytical Psychology and Weltanschauung. The Collected Works of C. G. Jung.

[9] Comte A. (2009). The Positive Philosophy of Auguste Comte.

[10] Hatfield G., Fox C., Porter R., Wokler R. (1995). Remaking the Science of Mind: Psychology as Natural Science. Inventing Human Science.

[11] Coon D.J. (1992). Testing the limits of sense and science: American experimental psychologists combat spiritualism, 1880–1920. Am. Psychol..

[12] James W. (1892). A plea for psychology as a “natural science”. Philos. Rev..

[13] Lewin K. (1935). A Dynamic Theory of Personality.

[14] Fuchs A.H., Milar K.S., Freedheim D.K., Weiner I.B. (2003). Psychology as a Science. Handbook of Psychology: Volume 1, History of Psychology.

[15] Costall A. (2006). “Introspectionism” and the mythical origins of scientific psychology. Conscious. Cognit..

[16] Kusch M. (1995). Psychologis.

[17] Dilthey W. (1989). Introduction to the Human Sciences.

[18] Scanlon J. (1989). Dilthey on psychology and epistemology. Hist. Philos. Q..

[19] Foucault M. (1991). Discipline and Punish.

[20] Jung C.G. (1946). Analytical Psychology and Education. The Collected Works of C. G. Jung.

[21] Weber M., Gerth H.H., Wright Mills C. (1946). Science as a Vocation. Max Weber: Essays in Sociology.

[22] Harrington A. (1999). Renenchanted Science.

[23] Freud S. (1965). New Introductory Lectures on Psycho-Analysis.

[24] Jung C.G. (1948). The Phenomenology of the Spirit in Fairytales. The Collected Works of C. G. Jung.

[25] James W. (1902). The Varieties of Religious Experience.

[26] Shamdasani S. (2003). Jung and the Making of Modern Psychology.

[27] Jung C.G. (1910). The Association Method. Am. J. Psychol..

[28] Piaget J. (1962). Play, Dreams and Imitation in Childhood.

[29] Allen C. (1942). A mystical psychology. Nature.

[30] Westmann H. (1954). Selections from the works of C.G. Jung. Nature.

[31] Jung C.G. (1961). Symbols and the Interpretation of Dreams. The Collected Works of C. G. Jung.

[32] Allen C. (1961). Jungian psychology. Nature.

[33] Rychlak J.F., Papadopoulous R.K., Saayman G.S. (1984). Jung as Dialectician and Teleologist. Jung in Modern Perspective.

[34] Bair D. (2003). Jung.

[35] Shamdasani S. (1998). Cult Fictions.

[36] Billig M. (1999). Freudian Repression.

[37] Pietikäinen P. (1999). C.G. Jung and the Psychology of Symbolic Forms.

[38] Popper K.R. (1958). The Logic of Scientific Discovery.

[39] Jones R.A. (2003). Mixed metaphors and narrative shifts: Archetypes. Theory Psychol..

[40] Brooke R. (1991). Jung and Phenomenology.

[41] Rowland S. (2005). Jung as Writer.

[42] Hillman J. (1983). Healing Fiction.

[43] Jung C.G. (1922). On the relation of analytical psychology to poetry. The Collected Works of C. G. Jung.

[44] Jung C.G. (1989). Analytical Psychology: Notes of the Seminar Given in 1925.

[45] Jung C.G. (1952). Symbols of Transformation. The Collected Works of C. G. Jung.

[46] Wittgenstein L. (1953). Philosophical Investigations.

[47] Jones R.A. (2004). The science and meaning of the self. J. Anal. Psychol..

